# Antibiotic-Resistant Bacteria, Antimicrobial Resistance Genes, and Antibiotic Residue in Food from Animal Sources: One Health Food Safety Concern

**DOI:** 10.3390/microorganisms11010161

**Published:** 2023-01-08

**Authors:** Muhammad Usman Qamar, Aatika, Muhammad Ismail Chughtai, Hasan Ejaz, Bi Bi Zainab Mazhari, Uzma Maqbool, Awadh Alanazi, Yasir Alruwaili, Kashaf Junaid

**Affiliations:** 1 Department of Microbiology, Faculty of Life Sciences, Government College University Faisalabad, Faisalabad 38000, Pakistan; 2 Food Safety Laboratories, Animal Sciences Division, Nuclear Institute for Agriculture and Biology (NIAB), Faisalabad 38950, Pakistan; 3 Department of Clinical Laboratory Sciences, College of Applied Medical Sciences, Jouf University, Sakaka 72388, Saudi Arabia; 4 Department of Clinical Laboratory Sciences, College of Applied Medical Sciences, Jouf University, Qurayyat 75911, Saudi Arabia; 5 School of Biological and Behavioral Sciences, Queen Mary University of London, London E1 4NS, UK

**Keywords:** antimicrobial resistance in food, antimicrobial resistance genes, antimicrobial residue, food samples, pathogenic microorganisms, tetracyclines

## Abstract

Antibiotic-resistant bacteria causing foodborne serious illnesses can be found in contaminated food. Therefore, this study aimed to identify the pathogens, genes, and antimicrobial residues present in raw milk and meat. We collected 40 raw milk and 40 beef samples using the aseptic method from various parts of the Faisalabad metropolis, Pakistan. The samples were cultured on blood, MacConkey, and UTI chrome agar. The VITEK 2 compact system was used for microbial identification and determination of minimum inhibitory concentrations. Antimicrobial resistance genes for extended-spectrum β-lactamases, methicillin resistance in *Staphylococcus aureus*, and carbapenem resistance were identified using molecular techniques. ELISA was used to determine the tetracycline residue level in each sample. The beef samples showed polymicrobial contamination with 64 bacterial isolates, with *Escherichia coli* (29; 45.3%) and *Klebsiella pneumoniae* (11; 17.1%) predominating. The milk samples showed polymicrobial contamination with 73 bacterial isolates, with *E. coli* (22; 30%), *K. pneumoniae* (12; 16.4%), and *S. aureus* (10; 13.6%) forming the majority. Twenty-eight (43.7%) isolates from beef harbored *tet* genes, nineteen (29.6%) *bla*_CTX-M_, and fourteen (21.8%) *bla*_NDM-1_, and twenty-six (35.6%) isolates from milk harbored *tet* genes, nineteen (26%) *bla*_TEM_ and *bla*_CTX-M_, and three (4%) *bla*_NDM-1_. Twenty-two (55%) each of the beef and milk samples exceeded the maximum residue limit for tetracycline. Polymicrobial contamination by bacteria possessing *bla*_CTX-M_, *bla*_TEM_, *bla*_NDM-1_, *bla*_OXA_, *mecA*, and *tet* genes was identified in food samples. The high tetracycline residue levels pose a serious health risk to consumers.

## 1. Introduction

The United Nations estimates that 9.7 billion people will live on the earth by 2050 and 10.9 billion by 2100 [1]. It is becoming increasingly difficult to guarantee that people can access safe, nourishing, and healthy food as the human population increases. Trace amounts of antibiotic residues can be found in meat, eggs, and milk as a result of the widespread use of antibiotics in food-producing animals for therapeutic, preventative, and growth-promoting purposes [2]. Animals or animal products produced under lax antibiotic regulations—without enforcement of drug withdrawal periods or residue testing programs—are sold in informal food markets in many low- and middle-income countries, including Pakistan [3]. Pakistan is an overpopulated nation with a growing demand for food sources that contain protein, which has led to the establishment of numerous dairy, poultry, beef, and egg producing operations across the nation [4].

Transmission of antibiotic-resistant bacteria (ARB) to humans, immunopathological effects, allergies, mutagenicity, nephropathy, hepatotoxicity, bone marrow toxicity, and even carcinogenicity (e.g., oxytetracycline) are just a few of the side effects of antibiotic residues [5]. One of the effective antibiotics frequently used in the veterinary field is tetracycline [6]. Depending on drug formulation, the recommended oxytetracycline withdrawal period is 4 days for milk and 5–20 days for meat [7,8]. The maximum residue limit (MRL) of tetracycline, oxytetracycline, and chlortetracycline in meat and milk is 100 ppb (µg/kg or µg/L) as described in European Union law [9]. Due to food producers’ lack of compliance with drug withdrawal periods, drug residues are common in food products [10]. Antimicrobial resistance (AMR) in the food chain has emerged as a global “One Health” concern [11,12]. Broiler chicken meat, eggs, and milk may contain antibiotic residues, which increase the likelihood of the presence of ARB, posing a serious threat to public health [13]. The most common food contaminants are *Escherichia coli*, *Salmonella* spp., *Campylobacter* spp., *Shigella* spp., *Proteus* spp., and *Staphylococcus aureus* [14]. Tetracyclines appear to be present at higher concentrations and detection frequencies in meat products than quinolones, while aminoglycosides and beta-lactams are also commonly detected. Antimicrobial resistance genes (ARGs) conferring resistance to tetracycline (*tet*), extended-spectrum β-lactamase (ESBL) genes (*bla*_CTX-M_ and *bla*_TEM_), carbapenem resistance genes (*bla*_OXA_ and *bla*_NDM_), and a methicillin resistance gene (*mecA*) are the most commonly detected in ARB present in food and food products and are thought to be widely prevalent in the environment due to the widespread use of the corresponding antibiotics [15,16,17,18]. This study aimed to identify bacterial pathogens, ARGs, and antimicrobial residues present in raw milk and meat and discusses the significance of bacterial contamination of food for the “One Health” approach and food safety and security in the twenty-first century.

## 2. Materials and Methods

### 2.1. Study Design

This study was conducted prospectively at Government College University in Faisalabad. The study adheres to the Declaration of Helsinki [19] on ethical research and does not involve human or animal subjects.

### 2.2. Sample Collection

**Figure 1:**
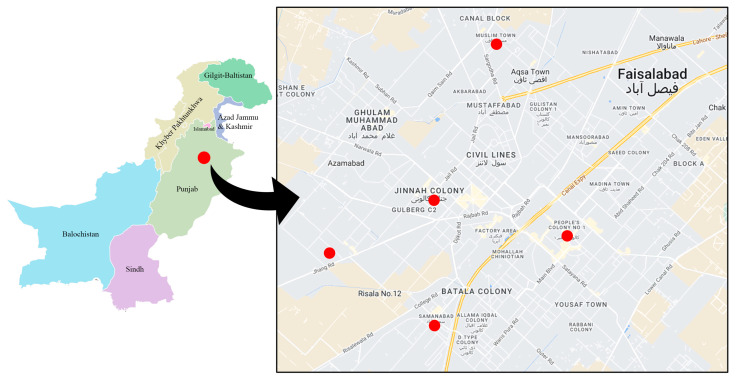
Map showing the sample collection sites in Faisalabad, Pakistan.

This was a cross-sectional study to discover how common AMR and antibiotic residues are in samples of animal products used to produce food. We obtained 40 samples of beef and 40 samples of milk from butcher markets and milk stores in five neighborhoods of the Faisalabad metropolitan area. These neighborhoods include Jinnah Colony, Shadab Colony, Samanabad Colony, People’s Colony, and Muslim Town Colony (Figure 1). Beef samples of 100–150 g and milk samples of 50–70 mL were collected in sterile bags and conical tubes, respectively. The samples were delivered to the microbiology laboratory in a container chilled to 4–6 °C for further examination.

### 2.3. Identification of Bacteria from Food Samples

Minced raw meat samples (25 g each) were cultured on nutrient agar, MacConkey agar, and UTI Chrome agar (Oxoid, Hampshire, UK). Milk samples (100 µL each) were serially diluted (10-fold) and cultured on nutrient agar, MacConkey agar, and UTI Chrome agar (Oxoid, Hampshire, UK), and then aerobically incubated overnight at 37 ºC. The isolates were preliminarily identified based on colony morphology, cultural characteristics, and Gram staining. The VITEK 2 compact instrument was employed to automatically identify isolates using GN cards (bioMérieux, Marcy-l’Étoile, France) with 64 different biochemical substrates.

### 2.4. Antimicrobial Susceptibility Testing (AST)

Bacteria isolated from beef and milk samples were tested for antimicrobial susceptibility with the VITEK 2 compact system, in accordance with Clinical and Laboratory Standards Institute (CLSI) 2022 guidelines [20]. Ampicillin, tetracycline, ciprofloxacin, amikacin, trimethoprim/sulfamethoxazole, co-amoxiclav, cefepime, ceftriaxone, colistin, cefoxitin, penicillin, ofloxacin, tobramycin, gentamicin, erythromycin, vancomycin, imipenem, and meropenem were among the antibiotics tested. The results were interpreted following the CLSI 2022 guidelines. *E. coli* ATCC 25922 was included to ensure the quality of AST [21].

### 2.5. Phenotypic Detection of Methicillin-Resistant Staphylococcus aureus

*Staphylococcus aureus* was phenotypically confirmed according to CLSI recommendations. A Mueller Hinton agar (MHA) plate was prepared, and a 0.5 McFarland bacterial suspension was lawned on it. An antibiotic disc containing cefoxitin (30 µg) was placed on the plate and incubated overnight at 37 ºC. *S. aureus* was classified as methicillin-resistant *S. aureus* (MRSA) if the zone of inhibition was less than 15 mm.

### 2.6. Phenotypic Detection of Carbapenemase Production

The modified Hodge test (MHT) was used to detect carbapenemase. A 0.5 McFarland *E. coli* ATCC 25922 suspension was prepared in sterile normal saline and diluted 1:10 in sterile normal saline. Mueller-Hinton agar (MHA) plates were lawned with the bacterial suspension and a meropenem (10 µg) disk was positioned in the middle. Isolates were streaked from the edge of the disk to the edge of the plate. If the isolates showed an indentation resembling a clover leaf, the MHT was deemed positive [22,23].

### 2.7. Phenotypic Detection of Metallo-β-Lactamases

The double-disk synergy test was used to identify metallo-β-lactamases. MHA plates were lawned with a 0.5 McFarland bacterial dilution. Then, two meropenem and two ertapenem discs were placed on the plate 25 mm apart. To one each of the meropenem (10 µg) and ertapenem (10 µg) disks, 10 μL of a 0.5 M ethylenediaminetetraacetic acid (EDTA) solution was added. When the zone of inhibition of the EDTA discs was 5 mm larger than that of the non-EDTA discs, MBL production was considered effective [24,25].

### 2.8. Molecular Detection of Antimicrobial Resistance Genes in Food Samples

A commercially available bacterial genomic DNA kit (Thermo Fisher Scientific, Waltham, MA, USA) was used to extract DNA from clinical isolates. DNA purity was determined using NanoDrop Spectrophotometer (Thermo Fisher Scientific, Waltham, MA, USA) at 260 and 280 nm wavelengths. Electrophoresis (Bio-Rad, Watford, UK) was used to determine the integrity of the DNA. The primers used are listed in Table 1.

We detected *bla*_NDM_ using initial denaturation at 95 °C for 1 min and then denaturation at 95 °C for 45 s, annealing at 58 °C for 45 s, extension at 72 °C for 1 min, and a final extension at 72 °C for 5 min. Multiplex PCR was performed for *bla*_OXA-51_, *bla*_OXA-23_, and *bla*_OXA-24_ genes with initial denaturation at 94 °C for 5 min, denaturation at 95 °C for 30 s, annealing at 52 °C for 40 s, extension at 72 °C for 50 s, and final extension at 72 °C for 6 min. The annealing temperature for *mecA*, *tet*, *bla*_CTXM_, and *bla*_TEM_ was 53 °C, 59 °C, and 63 °C, respectively.

### 2.9. Detection of Tetracycline Residue in Beef Samples

ELISA (RIDASCREEN, Biopharm, Eppelheim, Germany) was used to detect tetracycline residues in beef samples. In brief, 1 g each of minced and homogenized beef samples was added to 9 mL of 20 mM PBS in a 50 mL conical tube. After shaking, the samples were centrifuged at room temperature for 10 min at 4000× *g*. A new glass vial was filled with 1 mL of supernatant and 2 mL of n-hexane, and then each glass tube was vortexed for 10 s. The samples were then centrifuged at 4000× *g* at room temperature for 10 min. To develop the assay, 50 µL of the lower layer of n-hexane was used.

### 2.10. Detection of Tetracycline Residue in Milk Samples

ELISA (RIDASCREEN) was used to detect tetracycline residues in milk samples. In brief, 50 mL each of milk samples was centrifuged at 3000 g for 10 min at 10 °C. After centrifugation, the upper cream layer was removed with a Pasteur pipette, and the skimmed milk (50 µL) was diluted with the kit’s sample buffer 2 (450 µL). For assay development, 50 µL each of the milk samples was used per well. An ELISA plate reader (BioTek, Winooski, VT, USA) was used to measure absorbance at 450 nm. The concentration of residues was calculated using the following equation, as specified in the manual:

(1) $$\mathrm{Residue}\;\mathrm{concentration}=\;\frac{\mathrm{Absorbance}\;\mathrm{of}\;\mathrm{sample}/\mathrm{standard}}{\mathrm{Absorbance}\;\mathrm{of}\;\mathrm{zero}\;\mathrm{standard}}\;\times 100$$

## 3. Results

### 3.1. Identification of Isolates from Food Samples

For the analysis of food contaminants, beef and milk samples were obtained from five neighborhoods of the Faisalabad metropolitan area: Jinnah Colony (*n* = 8); Muslim Town Colony (*n* = 8); Samanabad Colony (*n* = 8); People’s Colony (*n* = 8); and Shadab Colony (*n* = 8) (Figure 1). Of the 64 bacterial isolates from the 40 beef samples, 15 (23.4%) were present as polymicrobial populations and 35 (54.6%) as monomicrobial populations. Six (9.37%) of the isolates were gram-positive cocci, with *S. aureus* predominating, while 58 (90.6%) were gram-negative bacteria, of which 29 (50%) were *E. coli*, 11 (18.9%) were *Klebsiella pneumoniae*, and 7 (12%) were *Salmonella* spp. Of the 73 bacterial isolates from the 40 milk samples, 66 (90.4%) were polymicrobial and 7 (9.6%) were monomicrobial populations. gram-positive cocci made up thirteen of the seventy-three (17.8%) isolates, of which ten (76.9%) were *S. aureus* and three (23.1%) were *Enterobacter cloacae*. gram-negative bacteria accounted for sixty (82.1%) isolates, of which twenty-two (36.6%) were *E. coli*, twelve (20%) were *K. pneumoniae,* and six (8.2%) were *Serratia marcescens* (Table 2).

### 3.2. Minimum Inhibitory Concentration of Antibiotics against Food Pathogens

For antimicrobial susceptibility testing, we used 19 antibiotic classes from the Access, Watch, and Reserve (AWaRe) categories of the World Health Organization (WHO). There were seven antibiotics in the Access category, eleven in Watch, and one in Reserve. All *E. coli* isolates from the beef samples were resistant to gentamicin and ampicillin, 79.3% to amoxicillin/clavulanic acid, and 58.6% to tetracycline. Of the *K. pneumoniae* isolates, 90.9% were resistant to tetracycline and gentamicin, 72.7% to cephalosporins (ceftriaxone and cefepime), and 51.7% to ofloxacin. *Salmonella* spp. were resistant to ampicillin (100%), co-trimoxazole (85.7%), and gentamicin (57.1%) (Table 3). *E. coli* isolates from milk were resistant to ampicillin, gentamicin, ciprofloxacin, and tetracycline (in that order, with highest resistance to ampicillin). In the case of *K. pneumoniae* isolates, 83.3% were resistant to gentamicin, 66.6% to co-trimoxazole, and 58.3% to tetracycline. Furthermore, gentamicin was found to be effective against 88.8% of the isolates, ciprofloxacin and amoxicillin/clavulanic acid against 77.6%, and tetracycline against only 44.4%. In general, vancomycin, colistin, and tigecycline were the most effective antibiotics (Table 4).

### 3.3. Phenotypic Confirmation of Antimicrobial Resistance Genes in Food Samples

**Figure 2:**
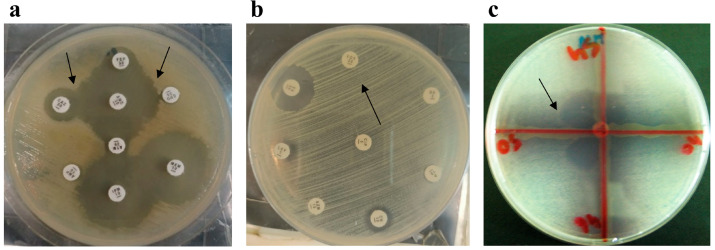
Phenotypic detection of antimicrobial resistance genes in isolates recovered from food samples. (**a**) Extended-spectrum β-lactamase-producing *E. coli*; the black arrows indicate the synergistic effect of antibiotics. (**b**) Methicillin-resistant *Staphylococcus aureus*; the black arrow indicates cefoxitin resistance. (**c**) Carbapenemase-producing *E. coli*; the black arrow indicates the extension of growth towards the meropenem disc.

Three phenotypic ARGs were found in the food samples. The following bacteria produced ESBLs in beef samples: nine (22.5%) *E. coli* isolates; four (10%) *K. pneumoniae* isolates; and one (2.5%) isolate each of *A. baumannii*, *P. aeruginosa*, and *E. cloacae*. Carbapenemase and MBL producers included four (10%) *E. coli* isolates, two (5%) *K. pneumoniae* isolates, and one (2.5%) isolate each of *E. cloacae* and *P. aeruginosa*. Three (7.5%) MRSA producers were also present. Eleven (27.5%) *E. coli* isolates, five (12.5%) *K. pneumoniae* isolates, three (7.5%) *S. marcescens* isolates, two (5%) isolates each of *A. baumannii* and *E. cloacae* produced ESBLs in milk samples, while three (7.5%) *E. coli* isolates also produced carbapenemase and MBLs. MRSA was present in three (7.5%) samples (Figure 2).

### 3.4. Genotypic Confirmation of Antimicrobial Resistance Genes in Food Samples

**Figure 3:**
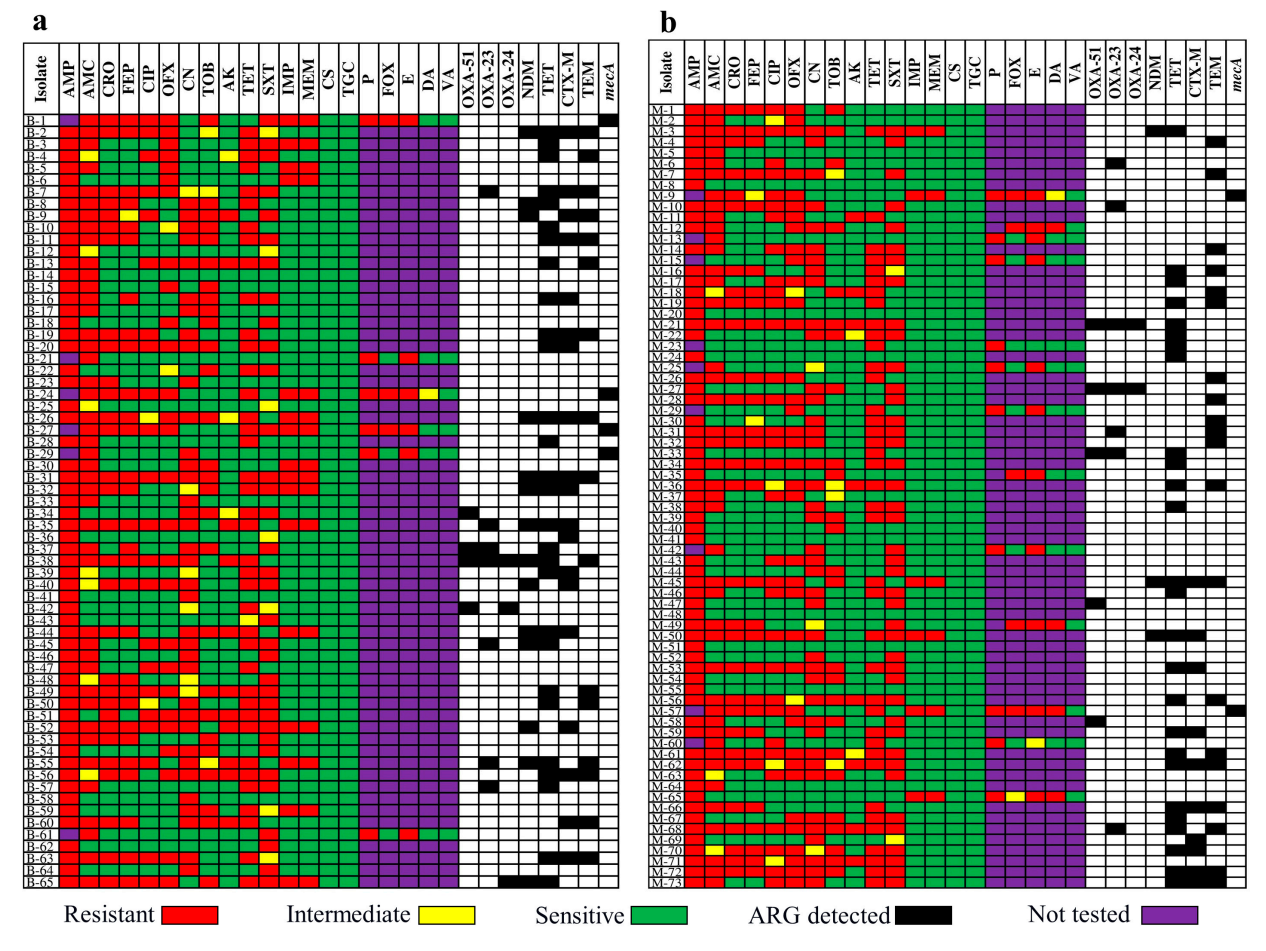
Coexistence of antimicrobial resistance (AMR) and antimicrobial resistance genes (ARGs) in different isolates. (**a**) AMR and AMGs in bacterial isolates of bovine. (**b**) AMR and AMGs in bacterial isolates of milk.

We examined eight ARGs for tetracycline resistance (*tet*), ESBL (*bla_CTX-M_* and *bla*_TEM_), carbapenem resistance (*bla*_NDM_, *bla*_OXA-23_, *bla*_OXA-24_, and *bla*_OXA-51_), and MRSA (*mecA*). The majority (twenty-eight [43.7%]) of the *tet* genes identified in the isolates from beef were detected in *E. coli*, six (21.4%) in *K. pneumoniae*, and three (10.7%) in *P. aeruginosa*. There were nineteen (29.6%) *bla*_CTX-M_ genes—eleven (57.8%) in *E. coli* and seven (36.8%) in *K. pneumoniae*. Furthermore, fifteen (23.4%) *bla*_TEM_ genes were found, with ten (66.6%) in *E. coli* and two (13.3%) in *K. pneumoniae*. Among the seven (10.9%) *bla*_OXA-23_ genes, two (28.7%) were in *E. coli* and two (28.7%) were in *P. aeruginosa*. Furthermore, fourteen (21.8%) *bla*_NDM-1_ genes were found, including six (42.8%) in *E. coli*, four (28.5%) in *K. pneumoniae*, and four (6.25%) in MRSA. Twenty-six (35.6%) *tet* genes were found in milk samples, with nine (34.6%) in *E. coli*, four (15.3%) in *K. pneumoniae*, and three (11.5%) in *P. aeruginosa*. Among the nineteen (26%) isolates that produced *bla*_TEM_, eleven (57.8%) were *E. coli*, three (15.7%) were *S. marcescens*, and three (15.7%) were *K. pneumoniae*. Nineteen (26%) isolates had *bla*_CTX-M_, including fourteen (73.6%) *E. coli* and four (21%) *K. pneumoniae*. Seven (9.5%) isolates harbored *bla*_OXA-23_, among them three (42.8%) isolates of *P. aeruginosa* and three (42.8%) of *A. baumannii*. *A. baumannii* produced two (2.7%) isolates with *bla*_OXA-24_ and five (6.8%) with *bla*_OXA-51_. Three (4%) *E. coli* isolates harbored *bla*_NDM-1_ and two (2.7%) *S. aureus* isolates harbored *mecA* (Figure 3).

### 3.5. Tetracycline Residues in Bovine Milk and Beef Samples

**Figure 4:**
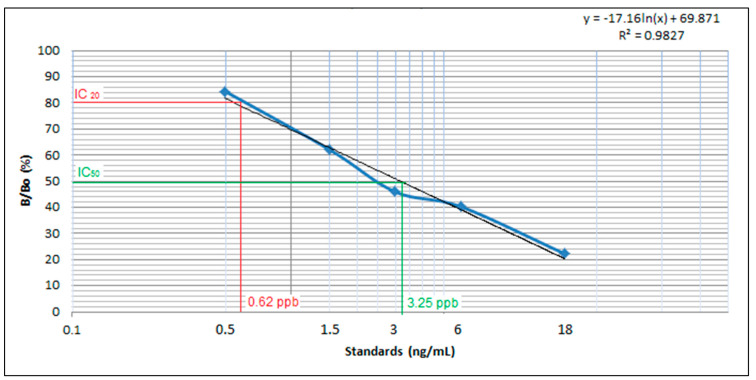
Calibration curve for tetracycline detection in beef muscle and milk by ELISA.

**Figure 5:**
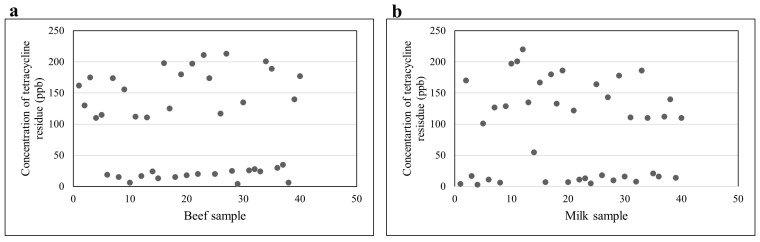
(**a**) Tetracycline residue in beef samples. (**b**) Tetracycline residue in bovine milk samples.

A commercial ELISA kit was used to determine the presence of tetracycline residues in bovine milk and beef samples. The kits were standardized and validated before screening using different standards, such as 0, 0.5, 1.5, 3, 6, and 18 ppb, as well as positive and negative controls. In order to calculate relative absorbance, optical density was measured at a wavelength of 450 nm. Relative absorbance (%) was used to construct a calibration curve (Figure 4). Based on the calibration curve, IC20 was calculated at 0.62 ppb and IC50 at 3.25 ppb. The calibration curve was interpolated to calculate the residue concentration in unknown samples. We analyzed 80 specimens (40 each of bovine milk and beef) collected from Faisalabad, Pakistan. Our results indicated that 22 of the 40 beef samples exceeded this limit, and had a residue concentration ranging from 110 to 213 ppb (Figure 5a). In our study, 22 of the 40 milk samples had residues above this limit, showing a concentration ranging from 101 to 220 ppb (Figure 5b).

### 3.6. Coexistence of Antimicrobial Resistance, Antimicrobial Resistance Genes, and Tetracycline Residues in Bovine Milk and Beef Samples

Twenty-two of the 40 (55%) beef samples had tetracycline residue levels greater than 100 ppb; of these, 17 samples had a *tet* gene and 21 were tetracycline-resistant. Twenty-two of the 40 (55%) milk samples also tested positive for tetracycline residue at >100 ppb; of these samples, 20 were tetracycline-resistant and 17 tested positive for the *tet* gene (Table 5).

## 4. Discussion

Public health is seriously threatened by food contamination by microbes. According to the WHO, contaminated food is responsible for about 600 million cases of illness and 420,000 deaths each year. The WHO estimates that approximately 1 in 10 cases of diarrhea worldwide is caused by tainted food [26]. The rising demand for meat protein around the world is correlated with the rising demand for poultry meat. For instance, the demand for poultry meat in South Asia is expected to rise dramatically (by 725%) by 2030, particularly in nations like India and Pakistan. Foodborne illnesses frequently occur when adequate attention is not paid to food hygiene and safety as food products move along the food chain. Food can become contaminated at different points in the production, distribution, and storage processes. Food safety is a more pressing issue in developing nations. In this study, we found evidence of polymicrobial contamination of samples of beef and milk from cows. *Salmonella* spp., *E. coli*, *K. pneumoniae*, and *S. aureus* were the most prevalent bacteria. Numerous studies have investigated the frequency of foodborne pathogens worldwide. A recent study found *S. aureus* and *Salmonella* spp. in samples of pork and chicken meat [27]. A previous Pakistani study of ready-to-eat food also found contamination with *E. coli*, *K. pneumoniae*, and *S. aureus* [28]. A study from Egypt found that 44.7% of bacterial isolates from food samples were *E. coli*, 17% were *Enterobacter* spp., and 12% were *Citrobacter* spp. [14]. In a study from Ethiopia, 52% of milk samples tested positive for *E. coli*, *K. pneumoniae*, *S. aureus*, and *Citrobacter* spp. in culture tests [29].

Antimicrobial resistance is a worldwide phenomenon induced by antimicrobial use (AMU). Global data indicate that AMU in food animals far outweighs that in human medicine. Although one of the main goals of the WHO’s Global Action Plan on AMR is to estimate AMU in food animals, there are few national-level estimates of veterinary antimicrobial use in low- and middle-income countries. Pakistan has developed a National Action Plan on AMR in response to the WHO’s Global Action Plan and is committed to addressing the AMR regulatory policy issue using the “One Health” approach. The broiler industry in Pakistan may use up to 568 tons of antimicrobials annually [30]. Various antibiotics are used in livestock for growth and disease control; however, the excessive use of these drugs in livestock leads to contamination of food with antimicrobial residues, which can have serious negative health effects on humans and animals [31]. Globally, and particularly in developing nations like Pakistan, AMR is becoming a serious threat to public health. This study found that milk and beef samples contained ESBL-producing bacteria, primarily *E. coli* and *K. pneumoniae* (harboring *bla*_CTX-M_ and *bla*_TEM_ genes), MRSA (with the presence of *mecA*), and carbapenem-resistant gram-negative rods (harboring *bla*_NDM-1_ and *bla*_OXA_ genes). *E. coli*, *K. pneumoniae*, *A. baumannii*, and *P. aeruginosa* are gram-negative bacteria resistant to various antibiotic classes, including β-lactams, fluoroquinolones, and tetracyclines; carbapenems and colistin were the most sensitive antibiotics. The most effective treatment for gram-positive bacteria (*S. aureus* and *Enterococcus* spp.) was vancomycin. Several previous studies from developing nations have reported findings that are essentially the same [32]. An almost identical finding of raw meat samples containing bacteria resistant to cephalosporins was made in a Polish study [33]. In addition, a study conducted in India discovered the presence of foodborne pathogens in raw milk samples [34]. Possible causes for this high level of milk contamination in the study area included the use of unpasteurized milk for commercial purposes, poor hygiene habits, insufficient cooling, and a lack of facilities that met standards for milking and storing and transporting milk. There is substantial evidence that microbial contamination in the milk market value chain can be caused by a sick cow, unhygienic milking techniques, bad personal hygiene, unsanitary utensils and/or milking equipment, unsanitary storage conditions, or the lack of a pure water supply.

Tetracyclines, effective against gram-positive and gram-negative bacteria, have a broad spectrum of activity and are widely used in livestock. They are used as growth promoters and as treatment for a variety of diseases in animals. The most commonly used antibiotics in livestock are tetracycline, oxytetracycline, chlortetracycline, and doxycycline [35]. According to European Union regulation, tetracycline residues in milk and meat should be no more than 100 g of tetracycline per kilogram [36]. The present study found 22 raw beef and 22 milk samples with >100 ppb of tetracycline residue. Our results are similar to those of an investigation from Lebanon that tested milk and dairy products for tetracycline residues [37]. A recent Tanzanian study also found meat samples with tetracycline residues [38]. A study from Kenya found that milk samples from the neighborhood market contained ARB and antibiotics [7]. Similarly, other studies have found antibiotic residues in animals used for food production [39]. Several factors facilitate the transmission of foodborne illnesses by food-producing animals, making it imperative to examine AMR, AMGs, and antibiotic residues in food-producing animals. This study has a few limitations, including the inability to detect all AMGs and MRLs for other antibiotic residues.

## 5. Conclusions

This study found polymicrobial contamination of food samples, specifically with *E. coli*, *K. pneumoniae*, *Salmonella* ssp., and MRSA. The majority of these bacteria possessed ARGs for ESBL (*bla*_CTX-M_ and *bla*_TEM_), carbapenem resistance (*bla*_NDM-1_ and *bla*_OXA_), and tetracycline (*tet* genes); MRSA harbored the *mecA* gene. More than half of the milk and beef samples contained tetracycline residues exceeding the MRL (>100 ppb)—the amount that can be ingested over a lifetime without causing any discernible health risk—requiring regulatory bodies to take immediate action according to the FAO/WHO Codex Alimentarius Commission’s instructions. Furthermore, the “One Health” approach should be adapted to combat antimicrobial residues, AMR, and AMU in animals, humans, and the environment.

## Figures and Tables

**Table 1:** Primers used for the identification of antimicrobial resistance genes.

Gene	Primer (5′ to 3′)	Annealing Temperature
*bla*_CTX-M_-F	ATGTGCAGYACCAGTAARGTKATGGC	62 °C
*bla*_CTX-M_-R	TGGGTRAARTARGTSACCAGAAYCAGCGG	
*bla*_TEM_-F	CGCCGCATACACTATTCTCAGAATGA	62 °C
*bla*_TEM_-R	ACGCTCACCGGCTCCAGATTTAT	
*bla*_NDM-1_-F	ATGGAATTGCCCAATATTATGCAC	58 °C
*bla*_NDM-1_-R	TCAGCGCAGCTTGTCGGC	
*bla*_OXA_-51-F	TAATGCTTTGATCGGCCTTG	52 °C
*bla*_OXA_-51-R	TGGATTGCACTTCATCTTGG	
*bla*_OXA_-23-F	GATCGGATTGGAGAACCAGA	52 °C
*bla*_OXA_-23-R	ATTTCTGACCGCATTTCCAT	
*bla*_OXA_-24-F	GGTTAGTTGGCCCCCTTAAA	52 °C
*bla*_OXA-24_-R	AGT TGAGCGAAAAGGGGATT	
*mecA*-F	AAAATCGATGGTAAAGGTTGGC	53 °C
*mecA*-R	AGTTCTGGAGTACCGGATTTGC	
*tet*-F	GGCCTCAATTTCCTGACG	59 °C
*tet*-R	AAGCAGGATGTAGCCTGTGC	

**Table 2:** Bacteria detected in beef and bovine milk samples collected from different locations in the Faisalabad metropolitan area, Pakistan.

Source of Sample	Beef Sample	Isolate	Milk Sample	Isolate
Jinnah Colony	B-1	*S. aureus*	M-1	*E. coli*
		*E. coli*		*Pseudomonas aeruginosa*
	B-2	*E. coli*	M-2	*E. coli*
	B-3	*Proteus mirabilis*	M-3	*K. pneumoniae*
		*E. coli*	M-4	*K. pneumoniae*
	B-4	*Salmonella* spp.		*P. aeruginosa*
		*E. coli*	M-5	*E. coli*
	B-5	*K. pneumoniae*		*Salmonella* spp.
	B-6	*E. coli*	M-6	*S. aureus*
	B-7	*E. coli*		*P. aeruginosa*
		*K. pneumoniae*	M-7	*K. pneumoniae*
	B-8	*E. coli*		*Enterococcus faecalis*
	B-9	*E. coli*	M-8	*S. aureus*
Muslim Colony	B-10	*E. coli*		*E. coli*
		*P. mirabilis*	M-9	*S. aureus*
	B-11	*K. pneumoniae*		*Serratia marcescens*
	B-12	*P. mirabilis*	M-10	*K. pneumoniae*
		*E. coli*		*E. coli*
	B-13	*E. coli*	M-11	*Enterobacter cloacae*
		*K. pneumoniae*		*E. coli*
	B-14	*S. aureus*	M-12	*Acinetobacter baumannii*
		*E. coli*		*E. coli*
	B-15	*E. coli*	M-13	*S. aureus*
		*S. aureus*		*E. coli*
	B-16	*Salmonella* spp.	M-14	*S. aureus*
		*E. coli*		*E. coli*
	B-17	*S. aureus*	M-15	*A. baumannii*
		*E. coli*		*E. coli*
	B-18	*S. aureus*	M-16	*S. aureus*
		*E. coli*		*S. marcescens*
Samanabad Colony	B-19	*E. coli*	M-17	*P. aeruginosa*
		*K. pneumoniae*		*E. coli*
	B-20	*E. coli*	M-18	*A. baumannii*
		*A. baumannii*		*E. coli*
	B-21	*E. coli*	M-19	*E. faecalis*
	B-22	*K. pneumoniae*		*K. pneumoniae*
		*Salmonella* spp.	M-20	*E. coli*
	B-23	*A. baumannii*	M-21	*E. coli*
		*E. coli*	M-22	*E. cloacae*
	B-24	*K. pneumoniae*		*S. marcescens*
	B-25	*E. coli*	M-23	*K. pneumoniae*
		*A. baumannii*		*S. aureus*
	B-26	*Salmonella* spp.	M-24	*Salmonella* spp.
	B-27	*E. coli*		*E. coli*
People’s Colony		*P. aeruginosa*	M-25	*E. coli*
	B-28	*E. cloacae*		*P. aeruginosa*
		*K. pneumoniae*	M-26	*A. baumannii*
	B-29	*E. coli*		*P. aeruginosa*
	B-30	*E. coli*	M-27	*E. faecalis*
		*E. cloacae*		*E. coli*
	B-31	*K. pneumoniae*	M-28	*E. coli*
		*E. cloacae*		*K. pneumoniae*
	B-32	*Salmonella* spp.	M-29	*K. pneumoniae*
	B-33	*E. coli*	M-30	*Citrobacter freundii*
	B-34	*K. pneumoniae*		*K. pneumoniae*
		*E. coli*	M-31	*P. aeruginosa*
	B-35	*P. aeruginosa*		*S. aureus*
	B-36	*E. coli*	M-32	*A. baumannii*
Shadab Colony		*Salmonella* spp.	M-33	*K. pneumoniae*
	B-37	*K. pneumoniae*		*S. aureus*
	B-38	*S. aureus*	M-34	*S. marcescens*
		*E. coli*		*E. coli*
	B-39	*E. coli*	M-35	*P. aeruginosa*
		*Salmonella* spp.	M-36	*S. marcescens*
	B-40	*P. aeruginosa*		*S. aureus*
	-	*-*	M-37	*E. coli*
	-	*-*		*P. aeruginosa*
	-	*-*	M-38	*E. coli*
	-	*-*		*Salmonella* spp.
	-	*-*	M-39	*S. marcescens*
	-	*-*		*K. pneumoniae*
	-	*-*	M-40	*E. coli*
	-	*-*		*K. pneumoniae*

**Table 3:** Antimicrobial resistance profile (minimum inhibitory concentration [µg/mL]) of bacteria isolated from beef samples.

Isolates from Beef	AMP (≥32)	CRO (≥4)	FEP (≥16)	AMC (≥32/16)	CIP (≥4)	OFX (≥2)	TET (≥16)	TOB (≥16)	AK (≥64)	CN (≥16)	SXT (≥4/76)	IMP (≥4)	MEM (≥4)	CS (≥4)	P (≥0.25)	E (≥8)	FOX (≥8)	DA (≥4)	VA (≥16)
WHO classification	Access	Watch	Watch	Access	Watch	Watch	Watch	Watch	Access	Access	Access	Watch	Watch	Reserve	Access	Watch	Watch	Access	Watch
*Escherichia coli* (*n* = 29)	100%	44.8%s	33.0%	79.3%	44.8%	51.7%	58.6%	44.8%	20.8%	62%	55.1%	24.1%	20.6%%	0%	NT	NT	NT	NT	NT
*Klebsiella pneumoniae* (*n* = 11)	NT	72.70%	72.7%	72.7%	45.5%	36.3%	90.9%	72.7%	27.2%	90.9%	63.6%	18.1%	18.1%	0%	NT	NT	NT	NT	NT
*Salmonella* spp. (*n* = 7)	100%	0%	0%	28.5%	0%	14.2%	28.7%	28.7%	0%	57.1%	85.7%	0%	0%	NT	NT	NT	NT	NT	NT
*Acinetobacter baumannii* (*n* = 3)	100%	33.3%	33.3%	33.3%	33.3%	33.3%	100%	33.3%	33.3%	100%	66.6%	0%	0%	0%	NT	NT	NT	NT	NT
*Enterobacter cloacae* (*n* = 3)	100%	66.6%	66.6%	66.6%	66.6%	33.3%	66.6%	100%	33.3%	100%	100%	33.3%	33.3%	0%	NT	NT	NT	NT	NT
*Pseudomonas aeruginosa* (*n* = 3)	100%	66.6%	66.6%	100%	66.6%	66.6%	100%	33.3%	0%	33.3%	100%	33.3%	33.3%	0%	NT	NT	NT	NT	NT
*Proteus mirabilis* (*n =* 3)	100%	0%	0%	100%	33.3%	33.3%	33.3%	33.3%	0%	33.3%	33.3%	0%	0%	100%	NT	NT	NT	NT	NT
*Staphylococcus aureus* (*n* = 6)	NT	50%	50%	100%	50%	50%	33.3%	16.6%	0%	16.6%	50%	50%	50%	NT	100%	83.3%	50%	33.3%	0%

AMP, ampicillin; CRO, ceftriaxone; FEP, cefepime; AMC, co-amoxiclav; CIP, ciprofloxacin; OFX, ofloxacin; TET, tetracycline; TOB, tobramycin; AK, amikacin; CN, gentamicin; SXT, trimethoprim/sulfamethoxazole; IMP, imipenem; MEM, meropenem; CS, colistin; P, penicillin; E, erythromycin; FOX, cefoxitin; DA, clindamycin; VA, vancomycin; NT, not tested.

**Table 4:** Antimicrobial resistance profile (minimum inhibitory concentration [µg/mL]) of bacteria isolated from bovine milk samples.

Milk Isolates	AMP (≥32)	CRO (≥4)	FEP (≥16)	AMC (≥32/16)	CIP (≥4)	OFX (≥2)	TET (≥16)	TOB (≥16)	AK (≥64)	CN (≥16)	SXT (≥4/76)	IMP (≥4)	MEM (≥4)	CS (≥4)	P (≥0.25)	E (≥8)	FOX (≥8)	DA (≥4)	VA (≥16)
WHO classification	Access	Watch	Watch	Access	Watch	Watch	Watch	Watch	Access	Access	Access	Watch	Watch	Reserve	Access	Watch	Watch	Access	Watch
*Escherichia coli* (*n* = 22)	100%	63.6%	63.6%	77.20%	68.1%	68.1%	63.6%	54.5%	18.1%	90.9%	59%	13.6%	13.6%	0%	NT	NT	NT	NT	NT
*Klebsiella pneumoniae* (*n* = 12)	NT	41.6%	41.6%	75%	50%	50%	58.3%	25%	16.6%	83.3 %	66.6%	0%	0%	0%	NT	NT	NT	NT	NT
*Pseudomonas aeruginosa* (*n* = 9)	100%	33.3%	33.3%	77.6%	77.6%	77.7%	44.4%	44.4%	11.10%	88.8%	66.6%	0%	0%	0%	NT	NT	NT	NT	NT
*Serratia marcescens* (*n* = 6)	100%	50%	66.6%	66.6%	33.3%	33.3%	66.6%	50%	16.6%	83.30%	66.6%	0%	0%	0%	NT	NT	NT	NT	NT
*Acinetobacter baumannii* (*n* = 5)	100%	60%	60%	60%	40%	40%	60%	60%	20%	80%	80%	0%	0%	0%	NT	NT	NT	NT	NT
*Salmonella* spp. (*n* = 3)	100%	0%	0%	0%	33.3%	33.3%	0%	0%	0%	33.3%	66.6%	0%	0%	0%	NT	NT	NT	NT	NT
*Enterobacter cloacae* (*n* = 2)	100%	100%	50%	100%	50%	50%	50%	50%	0%	50%	50%	0%	0%	0%	NT	NT	NT	NT	NT
*Citrobacter freundii* (*n* = 1)	100%	0%	0%	100%	0%	0%	0%	0%	0%	100%	0%	0%	0%	0%	100%	83.3%	50%	33.3%	0%
*Staphylococcus aureus* (*n* = 10)	NT	30%	30%	70%	30%	30%	50%	60%	0%	40%	50%	30%	30%	NT	100%	70%	30%	40%	0%
*Enterococcus faecalis* (*n* = 3)	100%	100%	100%	100%	33.30%	33,3%	0%	100%	66.6%	100%	66.6%	0%	0%	NT	NT	66.6%	100%	66.6%	0%

AMP, ampicillin; CRO, ceftriaxone; FEP, cefepime; AMC, co-amoxiclav; CIP, ciprofloxacin; OFX, ofloxacin; TET, tetracycline; TOB, tobramycin; AK, amikacin; CN, gentamicin; SXT, trimethoprim/sulfamethoxazole; IMP, imipenem; MEM, meropenem; CS, colistin; P, penicillin; E, erythromycin; FOX, cefoxitin; DA, clindamycin; VA, vancomycin; NT, not t.

**Table 5:** Coexistence of antimicrobial resistance, antimicrobial resistance genes, and tetracycline residues in bovine milk and beef samples.

Beef Sample	TET AR (ppb)	MRL (ppb)	TET Antibiotic	*tet* Gene	Milk Sample	TET AR (ppb)	TET Antibiotic	MRL (ppb)	*tet* Gene
B-1	162	100	R	Post	M-1	4	S	100	Neg
B-2	130	100	R	Post	M-2	170	R	100	Post
B-3	175	100	R	Post	M-3	17	S	100	Neg
B-4	110	100	R	Neg	M -4	3	S	100	Neg
B-5	115	100	R	Post	M-5	101	S	100	Neg
B-6	19	100	S	Neg	M-6	11	S	100	Neg
B-7	174	100	R	Post	M-7	127	R	100	Neg
B-8	15	100	S	Neg	M-8	6	S	100	Neg
B-9	156	100	R	Post	M-9	129	R	100	Neg
B-10	6	100	S	Neg	M-10	197	R	100	Post
B-11	112	100	R	Post	M-11	201	R	100	Post
B-12	17	100	S	Neg	M-12	220	R	100	Post
B-13	111	100	R	Post	M-13	135	S	100	Post
B-14	24	100	S	Neg	M-14	55	S	100	Neg
B-15	13	100	S	Neg	M-15	167	R	100	Neg
B-16	198	100	S	Neg	M-16	7	S	100	Neg
B-17	125	100	R	Neg	M-17	180	R	100	Neg
B-18	15	100	S	Neg	M-18	133	R	100	Post
B-19	180	100	R	Post	M-19	186	R	100	Post
B-20	18	100	R	Post	M-20	7	S	100	Neg
B-21	197	100	R	Post	M-21	122	R	100	Post
B-22	20	100	S	Neg	M-22	11	S	100	Neg
B-23	211	100	R	Post	M-23	13	S	100	Neg
B-24	174	100	R	Neg	M-24	5	S	100	Neg
B-25	20	100	S	Neg	M-25	164	R	100	Post
B-26	117	100	R	Neg	M-26	18	S	100	Neg
B-27	213	100	R	Post	M-27	143	R	100	Post
B-28	25	100	S	Neg	M-28	10	S	100	Neg
B-29	4	100	S	Neg	M-29	178	R	100	Post
B-30	135	100	R	Post	M-30	16	S	100	Neg
B-31	26	100	R	Neg	M-31	111	R	100	Post
B-32	28	100	S	Neg	M-32	8	S	100	Neg
B-33	24	100	S	Neg	M-33	186	R	100	Post
B-34	201	100	R	Post	M-34	110	R	100	Post
B-35	189	100	R	Post	M-35	21	S	100	Neg
B-36	30	100	S	Neg	M-36	16	S	100	Neg
B-37	35	100	R	Neg	M-37	112	R	100	Post
B-38	6	100	S	Neg	M-38	140	R	100	Post
B-39	140	100	R	Post	M-39	14	R	100	Post
B-40	177	100	R	Post	M-40	110	R	100	Post

AR: Antimicrobial residue; MRL: maximum residue limit; Neg: negative; Post, positive; R, resistant; S, sensitive; TET: tetracycline.

## Data Availability

All data are available within the manuscript.
